# Biodegradable Polymers and Textiles

**DOI:** 10.3390/jfb16010026

**Published:** 2025-01-15

**Authors:** Sandra Varnaitė-Žuravliova, Julija Baltušnikaitė-Guzaitienė

**Affiliations:** Department of Textile Technologies, Center for Physical Sciences and Technology, Demokratu Str. 53, LT-48485 Kaunas, Lithuania; julija.baltusnikaite@ftmc.lt

The increasing interest in developing biodegradable polymers through chemical treatments, microorganisms, and enzymes highlights a commitment to environmentally friendly disposal methods. These polymers are engineered to decompose within a specific period, transforming into products that can be easily discarded. They are sourced from a variety of waste materials and bioresources, including food and agricultural waste, starch, and cellulose. Typically, bioplastics made from renewable resources are more cost-effective than those derived from microbial sources, encouraging manufacturers to prioritize these options [1,2].

In general, polymers are classified into natural (polysaccharides and proteins) and synthetic (amides, esters, ethers, urethanes) biodegradable polymers, and synthetic-biopolymers (or hybrid systems) [1]. Biodegradable polymers (also called biopolymers) are materials designed to function for a defined period before breaking down into easily disposable products through a controlled process [2].

The environmental benefits of biodegradable polymers are significant. They contribute to the regeneration of raw materials, promote biodegradation, and help reduce carbon dioxide emissions, which are major contributors to global warming. Key factors influencing the biodegradation process include the material’s composition, polymer morphology, structural characteristics, and molecular weight [1,2].

Both natural and synthetic biodegradable polymers are being developed to meet specific physical and chemical requirements across various applications, such as cosmetics, coatings, wound dressings, and gene delivery. Furthermore, modified biopolymers are gaining traction in advanced fields like nanotechnology, medical implants, prosthetics, cryopreservation, and sanitation products, including surgical sutures, indicating their versatile potential for addressing environmental challenges [1,2].

The study by El-Fakharany, E. M. et al. [3] highlights the promising antiviral properties of bacterial polymeric silk produced by Bacillus sp. strain NE (a petroleum-originated bacteria), specifically composed of fibroin and sericin proteins. These proteins demonstrated significant inhibitory effects against various viruses, including herpes simplex virus type 1 (HSV1), adenovirus type 7 (ADV7), and hepatitis C virus (HCV). The research found that these proteins effectively block viral entry and replication, with specific IC50 values (representing the concentration at which 50% inhibition of cell lysis occurs) indicating their potency. Additionally, the bacterial silk proteins showed a reduction in reactive oxygen species (ROS) generation in HCV-infected cells, further supporting their antiviral efficacy.

The research [3] underscores the potential of bacterial silk proteins as a biopharmaceutical candidate due to their ability to be produced in large quantities at low cost, offering a sustainable option for antiviral drug development. These findings suggest that bacterial silk proteins could play a crucial role in managing viral infections, including potential applications in controlling COVID-19, either alone or in combination with other antiviral drugs. The high safety profile on normal cells also supports their consideration for medicinal applications.

The investigation by Tyubaeva, P. et al. [4] focuses on the impact of natural and synthetic porphyrins, specifically those containing the same metal atom, on the structure and properties of poly(-3-hydroxybutyrate) (PHB) in electrospun fibrous materials. The research highlights the potential of incorporating hemin (Hmi) and tetraphenylporphyrin with iron (Fe(TPP)Cl) into PHB to enhance its properties for biomedical applications. It was concluded that even small concentrations of porphyrins can significantly enhance the antimicrobial properties (by 12 times) and improve the physical and mechanical properties (by at least 3.5 times) of the PHB fibers. The additives affect the hydrophobicity of the materials, altering it by at least 5%. The study used various analytical techniques such as microscopy, X-ray diffraction, and differential scanning calorimetry to investigate the structural changes in the materials. The porphyrin complexes influence the supramolecular structure differently, affecting the crystalline and amorphous phases of PHB. The presence of trivalent iron in the tetrapyrrole ring helps achieve an optimal balance of electrical conductivity and viscosity for producing defect-free fibers.

These findings in the study [4] provide insights into designing PHB-based fibrous materials with tailored properties, including enhanced mechanical strength, antibacterial activity, and controlled wettability, through the strategic use of porphyrin additives.

The fashion industry, driven by consumerism and the rapid pace of trend cycles, has become a significant contributor to excessive consumption and textile waste. The demand for new products and materials is fueled by globalization and the increasing involvement of new designers. This has led to a need for more sustainable alternatives, especially in the face of growing concerns over environmental impacts. The study by da Silva Junior, C. J. G. et al. [5] presented highlights the potential of bacterial cellulose (BC)-based vegan leather as a sustainable option for fashion. The biopolymer, produced using plant-based natural dyes extracted from Allium cepa L., Punica granatum, and Eucalyptus globulus L and waterproofing agents from Melaleuca alternifolia essential oil and Copernicia prunifera wax, demonstrates impressive mechanical properties, including high tensile strength and flexibility, which are essential for durable fashion products. The material also exhibits promising water resistance, with minimal water interaction, making it a viable option for waterproof fashion. The research in [5] underscores the potential of BC as an alternative to traditional animal leather, offering both environmental and ethical benefits. By utilizing non-toxic, plant-based dyes, the process also reduces potential harm to both consumers and the environment. While the waterproofing process needs further refinement, the results of the investigations presented in [5] indicate a promising future for biotechnological materials in sustainable fashion. As consumer demand for eco-friendly options continues to grow, these innovations could lead to more efficient and cost-effective production methods, contributing to a shift towards sustainability in the fashion industry. Further research in this area will likely result in the continuous improvement of these materials, making them increasingly viable for large-scale production and adoption by both manufacturers and conscientious consumers.

Silver nanoparticles (AgNPs) possess remarkable plasmonic and antimicrobial properties, making them valuable for a wide range of industrial and consumer applications. However, concerns about their toxicity, even at low doses, necessitate the development of safer synthesis methods. Desai, A. S. et al. [6] explored the synthesis of AgNPs using plant extracts, focusing on turmeric and aloe vera, and assessed their physico-chemical properties, cytotoxicity, and wound-healing potential. Researchers in [6] revealed that AgNPs derived from turmeric extract exhibited superior wound-healing properties and the least toxicity in both in vitro and in vivo studies. The in vitro analysis, using HEK-293 cells and a dose-dependent Drosophila model, demonstrated that turmeric-extract-derived AgNPs promoted sustained cell growth without cytotoxic effects. Additionally, the in vivo study using Drosophila showed no adverse effects, such as climbing disability, even at high doses, highlighting the biocompatibility of these nanoparticles. The research also showed that the choice of plant extract plays a crucial role in the synthesis process and the resulting properties of the AgNPs. While aloe vera accelerated nanoparticle growth, it also led to the formation of various silver oxides, which could affect the overall efficacy. Thus, selecting the right plant extract is essential for optimizing the properties and biological interactions of AgNPs. The study in [6] suggested that turmeric-extract-derived AgNPs are a promising, environmentally friendly, and low-cost alternative to chemically synthesized silver nanoparticles. The biocompatibility and minimal cytotoxicity of turmeric-derived AgNPs make them an excellent candidate for further development and potential commercialization. With additional research into toxicity analysis and synthesis standardization, these nanoparticles could provide a safer, more sustainable option for various applications, particularly in the biomedical field.

Vonnie, J. M. et al. [7] successfully developed a biodegradable composite film made from cornstarch (CS) and eggshell powder (ESP) through a casting technique. The morphological and physicochemical properties of the ESP/CS film were thoroughly characterized, revealing a smooth, crack-free surface with a spherical and porous irregular shape, indicative of a large surface area. SEM and TEM analysis confirmed the uniform dispersion of ESP particles within the CS matrix and highlighted the phase separation, contributing to the film’s enhanced characteristics. The addition of ESP significantly improved several properties of the film compared to the pure CS film. The ESP/CS film exhibited enhanced moisture content, swelling power, water absorption, and water solubility. These improvements were attributed to the interactions between ESP and CS molecules, which strengthened the film structure. Fourier Transform Infrared Spectroscopy (FTIR) analysis confirmed the presence of carbonate minerals in the film and the formation of hydrogen bonds, further enhancing the film’s compactness and potential for adsorption applications. X-ray diffraction (XRD) analysis showed the semi-crystalline structure of the composite film, with an increased peak intensity, demonstrating the strong interactions between ESP and CS. The study in [7] also highlighted that ESP improved the mechanical strength, hardness, and barrier properties of the film without the need for cross-linking agents. The ESP/CS composite film, with its promising physicochemical and mechanical properties, can serve as an effective adsorbent for various target analytes, such as contaminants in food products, making it a potential candidate for sustainable and environmentally friendly applications in the food industry and other sectors.

Bio-polymer fibers, particularly alginate, have garnered significant attention over the past two decades due to their versatile applications in gene therapy, tissue engineering, wound healing, and controlled drug delivery. Alginate’s unique properties, such as its ease of sol–gel transformation, ion exchange capabilities, and acid stability, make it a highly valuable material in various fields. The study of Zdiri, K. et al. [8] has provided a comprehensive review of the modifications made to alginate fibers to enhance their properties, including the incorporation of nanoparticles, adhesive peptides, and both natural and synthetic polymers.

The authors in [8] highlighted the structure, source, and specific properties of alginate, as well as the various spinning techniques and the impact of solution parameters on the thermo-mechanical and physico-chemical properties of alginate fibers. The incorporation of fillers, such as nanoparticles, has proven to be an effective strategy for improving the physical, thermal, mechanical, and wound-healing properties of calcium alginate fibers, which hold substantial promise for specialized applications, particularly in the medical field. While the potential applications of alginate composite fibers are vast, including uses in cosmetics, sensors, drug delivery, tissue engineering, and water treatment, there remains considerable room for further research. Future studies could focus on the mixed spinning of alginate and other bio-polymers, such as chitosan, to explore new properties and applications. The continued development of innovative, sustainable materials made from alginate and other bio-polymers is crucial for expanding their use and enhancing their performance in medical and environmental applications. Overall, alginate fibers offer a promising, eco-friendly option for numerous advanced technologies, and their continued modification and optimization will likely lead to even more impactful applications in the future [8].

Egorov, A. et al. in their research [9] investigated various calcification methods for collagen and collagen coatings, assessing their suitability for 3D printing and the production of collagen-coated scaffolds. PCL scaffolds were printed and subsequently coated with collagen using four different methods: direct addition of hydroxyapatite (HA) powder to the collagen solution, incubation in Simulated Body Fluid (SBF), coating with alkaline phosphatase (ALP), and coating with poly-L-aspartic acid (poly ASP). The results of these methods were analyzed using ESEM, CT, TEM, and EDX.

The authors of [9] also found that the direct addition of HA powder to the collagen solution caused a pH shift and an increase in viscosity, leading to clumping on the scaffold surface. In contrast, incubation in SBF and with ALP resulted in an increase in HA layer thickness, but no coating was apparent on the collagen layer with poly-L-aspartic acid. Interestingly, only the poly-L-aspartic acid treatment led to the formation of nanocrystalline HA within the collagen layer, as detected by ultrathin sections and TEM with SuperEDX. Overall, the project demonstrated that HA layers could form at the surface of collagen-coated PCL scaffolds with most calcification methods, except for poly-L-aspartic acid. However, poly-L-aspartic acid treatment uniquely facilitated the incorporation of HA crystals within the collagen layer, offering a promising approach for enhancing the properties of collagen-based scaffolds for applications in tissue engineering and regenerative medicine. These findings contribute to a better understanding of the methods for producing collagen-coated scaffolds with controlled HA incorporation, which can be optimized for future biomedical applications [9].

Medical gloves have long been essential in protecting healthcare workers and individuals from infectious microorganisms and hazardous substances, playing a pivotal role in infection prevention. During the COVID-19 pandemic, their importance was further emphasized as they became widespread preventive tools. Lovato M. J et al. in their review [10] highlighted the evolution of medical gloves, beginning with the discovery of vulcanization by Charles Goodyear in 1839, which laid the foundation for the industry’s development. The paper [10] compared the properties, benefits, and drawbacks of various commonly used gloves, including those made from natural rubber (NR), polyisoprene (IR), acrylonitrile butadiene rubber (NBR), polychloroprene (CR), polyethylene (PE), and poly(vinyl chloride) (PVC). Additionally, it addressed the environmental impacts of conventional rubber glove production and discussed mitigation strategies such as bioremediation and rubber recycling. To improve glove performance, the review [10] examined the potential of biopolymers, biodegradable fillers, reinforcing fillers, and antimicrobial agents. These materials, such as poly(vinyl alcohol), starch, cellulose, chitin, and silica, offer enhanced properties for medical gloves, including biodegradability, antimicrobial effects, and improved mechanical characteristics. The incorporation of antimicrobial agents, including biguanides, quaternary ammonium salts, and metal nanoparticles (e.g., silver, copper, and zinc oxide), has also shown promise in combating drug-resistant bacteria and preventing cross-contamination. The review [10] further emphasized the importance of sustainability in medical glove production, particularly in terms of environmental, social, and financial impacts. Using performance-enhancing materials such as bio-fillers and biodegradable polymers presents a viable path to creating new gloves with better properties without compromising functionality. The integration of these materials into rubber formulations or through additional dipping processes offers a cost-effective and scalable solution for the industry. It was summarized in a review [10] that the continued innovation in medical glove production, including the optimization of manufacturing processes and the integration of advanced materials, will ensure the development of high-quality, sustainable, and effective gloves. These gloves will not only meet regulatory standards but also cater to the growing demand for performance-enhanced personal protective equipment.

Celestino, M. F. et al. in their study [11] successfully developed scaffolds based on poly(hydroxybutyrate) (PHB) and micronized bacterial cellulose (BC) through 3D printing. The incorporation of varying concentrations of micronized BC (0.25%, 0.50%, 1.00%, and 2.00%) into the PHB matrix allowed for the production of biocomposite filaments that predominantly retained the functional groups of PHB, as confirmed by Fourier Transform Infrared Spectroscopy (FTIR). Thermogravimetric analyses (TG and DTG) indicated that increasing the BC concentration lowered the peak temperature of PHB degradation, with the lowest degradation temperature observed in the filament with the highest BC concentration (PHB/2.0% BC). However, the thermal variation did not hinder the 3D printing process, as the melting temperature of PHB remained above the degradation points. The biological assays showed that the PHB/BC scaffolds were non-cytotoxic and provided suitable cell anchorage sites, suggesting their potential for use in tissue engineering applications. Additionally, the optimal micronization of BC at 20 Hz was found to offer the best granulometry and homogeneity for producing biocomposite filaments. X-ray diffraction (XRD) analysis revealed that BC micronized at this frequency maintained a balanced crystalline and amorphous structure, which is crucial for tissue regeneration as it allows BC to function as a reinforcing agent while also being biodegradable. The characterization techniques, including TGA, FTIR, and SEM, confirmed the structural integrity of the filaments and their suitability for 3D printing, with no significant thermal stability issues for the intended application. Moreover, the use of BC industrial scraps in the production of these filaments aligns with the principles of circular economy and sustainability, adding value to waste materials.

Overall, the results of the research outlined in Ref. [11] demonstrate the promising potential of PHB/BC-based biocomposite filaments for 3D printing applications in tissue engineering. The successful integration of BC micronization and the resulting biocomposite filaments not only highlight their mechanical and biological advantages but also contribute to sustainable practices in material development. Further studies will be needed to explore the full potential of these materials in clinical applications.

In the review [12] by Varnaitė-Žuravliova, S. and Baltušnikaitė-Guzaitienė, J., the authors summarized that regenerated cellulose fibers (RCFs) offer a versatile and highly valuable biomaterial with a range of medical applications due to their biocompatibility, biodegradability, and mechanical strength. These fibers are widely utilized in wound care, tissue engineering, and absorbable sutures, making them essential for medical textiles. RCFs help create moist environments conducive to healing in wound care, support cell attachment in tissue engineering, and eliminate the need for removal in suturing due to their biodegradability. Additionally, their use in surgical garments and diagnostic materials further highlights their importance in the medical sector. As the field progresses, the future of RCFs in medical textiles will be shaped by advancements aimed at improving production processes, such as closed-loop systems for solvent recovery and reducing environmental impact. Furthermore, the integration of nanotechnology promises to enhance the properties of RCFs, including antimicrobial activity, increased surface area, and improved mechanical strength. However, challenges remain in terms of the long-term clinical performance of RCFs, especially in complex medical applications like tissue scaffolds and implants. Research focusing on their degradation rates, mechanical stability, and tissue regeneration over extended periods will be crucial for advancing their use in these areas. There is also potential in developing reinforced RCF composites that can withstand higher mechanical stresses while maintaining biodegradability, which would enable new applications in fields such as orthopedics and cardiovascular surgery. Additionally, the functionalization of RCFs with antimicrobial agents or drug-loaded fibers holds promise, though cost-effective manufacturing processes for large-scale production need to be established to ensure affordability in global healthcare markets. The development of smart textiles incorporating RCFs and biosensors presents exciting opportunities, but challenges regarding scalability, durability, and cost remain. Furthermore, the regulatory approval process for RCF-based medical products, particularly in innovative areas like tissue engineering and drug delivery systems, requires clearer frameworks to accelerate commercialization [12].

Overall, while regenerated cellulose fibers offer immense potential for medical applications, further research, technological advancements, and regulatory clarity are essential to fully realize their capabilities and to ensure they can be effectively utilized in a wide range of medical contexts [12].

This Special Issue with 10 papers covers reviews and investigations made in the areas of biodegradable polymers, especially natural macromolecules, and textiles. Hopefully, all contributions will bring practical benefits and encourage further research in this field.

We would like to express our sincere thanks to the authors, reviewers, and the MDPI *JFB* editors, along with their teams, for their invaluable contributions.
